# Measuring medically unjustified hospitalizations in Switzerland

**DOI:** 10.1186/s12913-022-07569-3

**Published:** 2022-02-07

**Authors:** Yves Eggli, Patricia Halfon, Romain Piaget-Rossel, Thomas Bischoff

**Affiliations:** Primary Care and Public Health Center (Unisanté), University of Lausanne, Route de la Corniche 10, 1010 Lausanne, Switzerland

**Keywords:** Regional health planning, Patient admission, Continuity of patient care, Medicine, Pediatrics

## Abstract

**Background:**

Inappropriate use of acute hospital beds is a major topic in health politics. We present here a new approach to measure unnecessary hospitalizations in Medicine and Pediatrics.

**Methods:**

The necessity of a hospital admission was determined using explicit criteria related to the recorded diagnoses. Two indicators (i.e. “unjustified” and “sometimes justified” stays) were applied to more than 800,000 hospital stays and a random sample of 200 of them was analyzed by two clinicians, using routine data available in medical statistics. The validation of the indicators focused on their precision, validity and adjustment, as well as their usefulness (i.e. interest and risk of abuse).

**Results:**

Rates, adjusted for case mix (i.e. age of patient, admission planned or not), showed statistically significant differences among hospitals. Only 6.5% of false positives were observed for “unjustified stays” and 17% for “sometimes justified stays”. Respectively 7 and 12% of stays had an unknown status, due to a lack of sufficiently precise data. Considering true positives only, almost one third of medical and pediatric stays were classified as not strictly justified from a medical point of view in Switzerland. Among these stays, about one fifth could have probably been avoided without risk. To enable a larger ambulatory shift, recommendations were made to strengthen the ambulatory care, notably regarding post-emergency follow-up, cardiac and pulmonary functions’ monitoring, pain management, falls prevention, and specialized at-home services that should be offered.

**Conclusion:**

We recommend using “unjustified stays” and “sometimes justified stays” indicators to monitor inappropriate hospitalizations. The latter could help the planning of reinforced ambulatory care measures to pursue the ambulatory shift. Nonetheless, we clearly advise against the use of these two indicators for hospitals financing purposes.

**Supplementary Information:**

The online version contains supplementary material available at 10.1186/s12913-022-07569-3.

## Background

Inappropriate use of acute hospital beds is a challenging issue that has several detrimental implications. Not only does it increase the health care costs (in Switzerland, a group of experts recently highlighted the importance of the ambulatory shift, to reduce these costs [1]), but hospitalization in itself might be harmful [2, 3].

In Switzerland, except for some surgical procedures only reimbursed as ambulatory care (varicose veins, hemorrhoids, menisci, etc.), hospitalizations are always reimbursed, regardless of whether the admission was justified [4]. Whereas several authors analyzed the potential move towards ambulatory surgery [5–8], little has been proposed to substitute hospitalizations by ambulatory care, especially in medicine and pediatrics.

To reduce the number of hospitalizations one can either prevent them by offering optimal ambulatory care or avoid unjustified ones. The Ambulatory Care Sensitive Conditions (ACSC) indicator enables to monitor the first aspect by screening stays that might have been prevented had the patient had access to adequate primary care services [9, 10]. This indicator has the advantage of being very easy to compute from routinely available hospital medical records. However, it suffers several limitations, including lack of sensitivity and specificity [11], poor clinical relevance [12], and low proportion of hospitalization actually preventable [13], its main drawback being that high rates of ACSC might be associated to differences in admission hospital practices rather than to the quality of ambulatory care [14].

To work on the second aspect of hospitalization’s reduction, one can use the Appropriateness Evaluation Protocol (AEP) [15], which focuses on the justification of hospital admissions. Despite several adaptations, this protocol fails to provide consistent rates of inappropriate hospitalizations [16–18]. It is based on a limited number of conditions related to patients and does not account for newly available alternative ambulatory services, such as outpatient intravenous therapy, home oxygen therapy, 24 h nursing care services, and home rehabilitation. Moreover, when confronted to an expert panel consensus, AEP’s accuracy has only been judged as “fair” [19]. These limitations and the fact that AEP requires a detailed and labor-intensive review of medical records might explain why the use of this protocol, which was extensive in many European countries during almost three decades [20], has decreased lately.

Another approach, based on routinely available hospital data, consists in identifying health conditions – defined by ICD 10 – that could be managed without emergency admission to an inpatient bed [21] (about one fifth of all emergency admissions in England [22]). Unfortunately, this approach fails to account for secondary diagnoses or interventions, which might justify hospitalizations. Moreover, it ignores elective hospital admissions that can sometimes be substituted by ambulatory care (e.g., to investigate non-severe conditions) and, thus, prevented.

In short, we are currently missing a tool to screen hospital admissions that are not justified from a medical point of view. Such a tool would enable to measure the frequency of ACSC without biases due to differences in admission policies. In addition, it would be useful to evaluate the share of hospitalizations that might be prevented by offering appropriate ambulatory care.

An innovative approach has been proposed by SQLape® classification of patients, which is based on multiple diagnostic and surgical categories [23, 24]. If a patient has at least one diagnosis or one surgical operation justifying the hospitalization, their admission is considered justified. Excluding surgical and obstetrical stays (which are always considered as justified), all the other stays are considered poorly justified and classified either as “unjustified” (i.e. when all diagnoses “almost never” justify a hospital admission per se) or “sometimes justified” (i.e. when at least one diagnosis “sometimes” justifies a hospitalization depending on the severity of the illness) [25]. The categorization of diagnoses was established on an empirical basis over several years, with an adjustment of the algorithms through a feedback loop by a dozen of hospitals in the French and Italian parts of Switzerland. However, the indicators have not been scientifically validated yet, hence this paper.

Using routinely available data, we assessed the strengths and limitations of the “unjustified stays” and “sometimes justified stays” indicators. Classically, a good indicator should be unbiased, precise and valid, i.e. adjusted for risk factors having a strong association with the outcome, providing statistically significant deviations among hospitals, without too much false positives or negatives (numerator), and with a proper eligible population (denominator) [26]. Baker and Chassin recently proposed to add two additional criteria to judge the usefulness of outcome indicators [27]: providers should be able to influence substantially the outcome and its use should have little chance of inducing unintended adverse consequences.

The objective of our article was to present our two innovative indicators and to validate them according to the above criteria. We did not put too much emphasize on avoiding false negatives since the intention was to provide a measure of unjustified stays that could potentially be avoided without too much dispute (minimum value). This criterion could be strengthened if needed.

We used Swiss hospital medical statistics to provide a validation based on representative and extensive results by hospital, as well a sample of hospitalization to assess the frequency of false positives from a clinical point of view.

## Methods

### Data

The source population included all hospitalizations recorded in the *Medical Statistics of Hospitals (Federal Statistical Office)* with a discharge occurring in the years 2014 to 2016 and a length of stay greater than 1 (i.e. different admission and discharge dates).

Hospitalizations with chemo- or radiotherapy, with a surgical intervention requiring a surgical theater, or related to obstetrics (i.e. delivery, abortion) were excluded using the standard SQLape® tool [23, 24]. Moreover, based on suggestions from the clinicians reviewing our tool, we additionally excluded:

- stays with an admission after 6 PM and a discharge the next day, considering the time required to avoid missing a high risk;
- newborn stays (less than one year), since it was impossible to evaluate the prognosis from the minimal data set;
- and elective hospitalization for alcohol use disorder (ICD-10 Z502 or ICD-9-CM 9462 codes).

After those exclusions, the studied population included 823,096 eligible stays (2014-2016).

### Screening “unjustified stays” and “sometimes justified stays”

A stay was considered “justified” if one of the following conditions was met [23]:

- at least one diagnosis classified as “almost always” requiring a hospitalization (premature birth, acute myocardial infarction, shock, pulmonary embolism, stroke, peritonitis, agranulocytosis for instance);
- more than two failures among following vital organs: respiratory (chronic respiratory failure), cerebral (degenerative disease of brain, dementia), cardiac (heart failure), hepatic (liver cirrhosis), hematologic (coagulation disorders), renal (chronic nephropathy, end stage renal disease) [28];
- cardiac dysrhythmia with cardiac failure;
- hallucination or delirium of a patient not living in a nursing home;
- pneumonia if children less than 7 years old or patients with significant comorbidities (cardiac congenital malformation, heart failure, other disease of large vessels, interstitial pulmonary disease, acquired immunodeficiency syndrome, other immune disorder).

A stay was considered as “unjustified” if all its associated conditions were classified as “almost never” requiring a hospitalization (Parkinson’s disease, migraine, anemia, psoriasis, thyroid disorders for instance). All other stays were classified as “sometimes justified” (called “more or less justified stays” in the SQLape® tool [24]). The list of conditions that “almost always”, “sometimes”, or “almost never” require a hospitalization is provided in Additional file 1.

### Review of cases screened

Two senior clinicians (PH, TB) reviewed independently a random sample of 200 “unjustified” hospitalizations and another one of 200 “sometimes justified” stays screened by the SQLape tool. PH has a long experience in hospital and ambulatory settings as internist. TB was in charge of academic and medical supervision of the unit of family medicine in the Primary Care and Public Health Center (Unisanté).

Available data were age, gender, hospital department (medicine, surgery, pediatrics, obstetrics, etc.), length of stay, main and secondary diagnoses, procedures and delay between admission and procedure dates. Data were printed as a case summary and presented in one or two pages by hospital stay. All data were anonymous and did not include any information enabling the identification of the individuals (no date of birth, ZIP code, hospitals, etc.) [29].

In a first round, the two senior clinicians reviewed all the stays independently and had to answer the two following questions:

- Question 1: From your point of view, was this hospital stay necessary in an ideal context? Possible answers: yes, no, I don’t know.
- Question 2: If the stay is not necessary, give the most probable cause of the hospital admission.

For question 2, the reviewer had to choose among an a-priori list of reasons that might have precluded home return (listed in Additional file 2). In a second round, all divergent judgments on the necessity of admission were discussed in depth until a consensus was reached or a decision to keep the divergence was taken. The aim of this discussion was to make sure both clinicians considered all possible situations corresponding to the combination of diagnoses and treatments. They were authorized to reach a consensus “I don’t know” if they both judged that the divergence was due to a lack of more detailed data (e.g. labs results, medication or social environment).

### Statistical analysis

Following de Mast [30] and Brennan [31], we assessed agreement between the two reviewers’ judgment (three categories: necessary, unnecessary, unknown) using uniform kappa rather than Cohen’s kappa [32]. The uniform kappa assumes a chance measurement independent of the categories being measured and, thus, a probability of agreement by chance constant and equal to the inverse of the number of categories (here 0.33).

Since the outcome from second round was obtained by consensus (i.e. no independency), we did not report kappa and simply computed the agreement percentage (i.e. number of identical judgements divided by the total of observations).

### Observed, expected rates and surplus estimation

Observed rates were obtained by dividing the numbers of cases (i.e. unjustified or sometimes justified stays) by the number of eligible stays as defined above.

Using data from years 2014-2016, we computed expected rates by strata of risk, i.e. by age classes and type of admission (programmed vs urgent), in a two steps procedure. First, we computed rates on the whole sample, which contained all Swiss hospitals. Second, we selected hospitals with observed rates lower than expected ones, among general health care hospitals (referred as K1-type hospitals [33]). Third, we computed new average expected rates based on these benchmark hospitals. We applied control limits to take into account the random variations of both observed and expected rates [34]. Outlying hospitals correspond to hospitals with observed rates exceeding maximal control limits at the 95% level (unilateral test).

Since hospitals may require more than 24 h to exclude a life-threatening condition or serve social needs (e.g. child abused protection), the target of avoiding all “unjustified” and “sometimes justified” stays is not desirable. Consequently, we estimated the surplus as the difference between observed and expected rates multiplied by the size of the eligible population.

## Results

In the first round, higher kappa was obtained for unjustified than for sometimes justified stays (0.70 vs 0.50). After discussion (i.e. after round 2), 86% of stays screened as unjustified were classified as unnecessary by both reviewers (Table 1). This proportion was slightly lower (71%) for “sometimes justified” stays. About 7.5% of “unjustified” stays remained difficult to evaluate (i.e. unknown status for both reviewers) and about 5.5% were false positive (i.e. necessary for both reviewers). For “sometimes justified” stays, these proportions were a bit higher (11.5% unknown; 16% false positive).

**Table 1:** Review of 200 stays screened as “unjustified” or “sometimes justified” stays

UNJUSTIFIED STAYS		First round			Kappa:	0.70	Second round		Agreement	0.99
		Reviewer 2					Reviewer 2			
			?	UN	N	%	?	UN	N	%
Reviewer 1	Unknown	?	2	12		7.0%	15			7.5%
	Unnecessary	UN		154	1	77.5%		172		86.0%
	Necessary	N	1	26	4	15.5%	1	1	11	6.5%
	Proportions	%	1.5%	96.0%	2.5%	100.0%	8.0%	86.5%	5.5%	100.0%
SOMETIMES JUSTIFIED STAYS		First round			Kappa:	0.50	Second round		Agreement:	0.98
		Reviewer 2					Reviewer 2			
			?	UN	N	%	?	UN	N	%
Reviewer 1	Unknown	?	4	18	7	14.5%	22	2		12.0%
	Unnecessary	UN	1	113	8	61.0%	1	141		71.0%
	Necessary	N	5	28	16	24.5%		2	32	17.0%
	Proportions	%	5.0%	79.5%	15.5%	100.0%	11.5%	72.5%	16.0%	100.0%

*Abbreviations*: *UN* unnecessary, *N* necessary,? = unknown

The reasons for precluding home return are given in Table 2. A quarter of unjustified stays had no severe diagnosis after investigation, but patients were admitted as a precaution. About 20% of patients were admitted for pain treatment. The third most frequent cause was lack of security at home (13.5%).

**Table 2:** Causes of hospitalizations screened as unjustified or sometimes justified

CAUSES OF HOSPITALIZATIONS	UNJUSTIFIED		SOMETIMES JUSTIFIED	
	N	%	N	%
Medically necessary (by both reviewers)	11	5.50%	32	16.00%
Monitoring for suspicion of serious illness	51	25.50%	20	10.00%
Treatment of pain	43	21.50%	31	15.50%
Lack of security at home	27	13.50%	30	15.00%
Bedridden or very fragile patient	10	5.00%	9	4.50%
Remoteness of patient’s residence	7	3.50%	18	9.00%
Lack of compliance of the patient	5	2.50%	5	2.50%
Programmed operation not performed	5	2.50%	5	2.50%
Specialized skills not available in ambulatory setting	5	2.50%		0.00%
Wound dressing or care	4	2.00%	2	1.00%
IV antibiotherapy	2	1.00%	7	3.50%
Other	2	1.00%	6	3.00%
Isolation of immuno-suppressed patient	1	0.50%	1	0.50%
Parenteral nutrition	0	0.00%	1	0.50%
Severity of illness unknown	4	2.00%	21	10.50%
Unknown	23	11.50%	12	6.00%
Total	200		200	

Unjustified and sometimes justified stays represented 17.8 and 24% of eligible admissions, respectively (Table 3). Proportions were higher among children and elective hospitalizations. Globally, the unjustified surplus represented 2.5% of the eligible stays against 3.3% for the sometimes justified one, leading to a total of about 6% of poorly justified stays.

**Table 3:** Unjustified and sometimes justified hospitalization rates (2014 to 2016)

Age (Years)	Pro- gram- med	Eligible stays		Unjustified stays		Sometimes justified stays		
			All hospitals	Reference hospitals	Surplus	All hospitals	Reference hospitals	Surplus
01-16	no	37,234	33.4%	32.1%	478	26.1%	25.2%	344
01-16	yes	5,685	46.4%	40.7%	322	21.4%	20.8%	33
16-50	no	158,202	26.1%	22.7%	5,271	26.4%	23.5%	4,622
16-50	yes	32,081	33.6%	27.4%	1,979	28.0%	27.3%	231
51-70	no	173,053	19.1%	16.2%	5,019	23.2%	19.8%	5,843
51-70	yes	37,443	29.8%	25.1%	1,750	27.6%	25.6%	749
71-80	no	139,520	11.0%	9.2%	2,459	23.2%	19.2%	5,538
71-80	yes	25,568	18.1%	15.9%	567	28.7%	24.6%	1,036
> 80	no	190,536	6.7%	5.4%	2,463	20.8%	16.6%	8,013
> 80	yes	23,774	10.7%	8.9%	438	25.3%	20.5%	1,139
Global		823,096	17.8%	15.3%	20,744	24.0%	20.6%	27,547
(proportion)					(2.52%)			(3.35%)

**Fig. 1:**
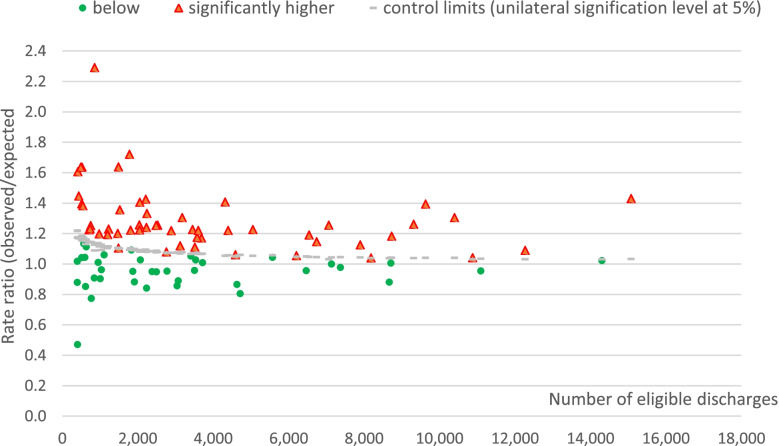
Unjustified rates ratios (observed/expected) by hospital, ranged by the number of eligible discharges

**Fig. 2:**
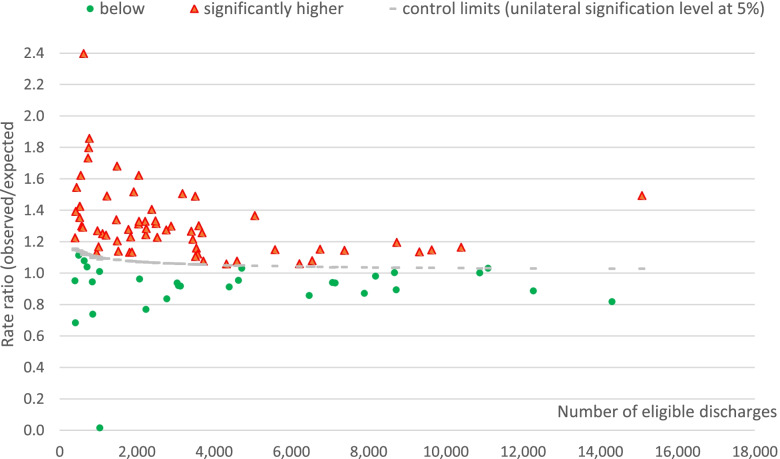
Sometimes justified rates ratios (observed/expected) by hospital, ranged by the number of eligible discharges

We plotted the ratios of observed divided by expected rates for unjustified and sometimes justified stays against the number of eligible discharges (Figs. 1 and 2). Only hospitals with at least one admission per day are shown. Control limits are given by grey lines, hospitals with observed rates significantly higher than maximum expected ratios are represented by (red) triangles, hospitals with lower values in (green) circles. Since the expected rate was computed using benchmark hospitals only, a majority of hospitals have higher rates than expected (i.e. ratio > 1.0). There are as many hospitals above and below control limits among large and smaller hospitals for both indicators.

Additional file 2 provides the list of all diagnoses involved in poorly justified hospitalizations, which might suggest some propositions for alternative ambulatory care (see Discussion below).

## Discussion

In this paper, we applied the indicators “unjustified stays” and “sometimes justified stays” to a random sample of 200 hospitalizations in Medicine and Pediatrics to estimate the proportion of those hospitalizations that were indeed unnecessary from an a posteriori medical point of view and to understand the motives of those admissions. Then, we applied these two indicators to all eligible hospitalizations in Switzerland from 2014 to 2016 (820,000 stays) to analyze the variability of the results among hospitals and to estimate the potential of hospitalization reduction.

Agreement between reviewers at the first round was rather good for unjustified stays, whereas it was only fair for “sometimes justified” stays. The main reason for these divergences was the lack of detailed clinical data, which lead the two reviewers to refer to different situations. The discussion of all possible stories improved the agreement among reviewers (kappa of 0.99 and 0.98 respectively). Two clinicians made medical judgements and involving more physicians would probably result in less agreement. The proportion of unknown status increased between the first and the second round to about 8% for “unjustified” and 12% for “sometimes justified” stays (Table 1). These results emphasize the main limitation of our study: the lack of details on patients (e.g. no information about the severity of illnesses, laboratory, and drugs). It must be emphasized that the reviewers had much more precise information (more than 16,000 diagnostic codes and 11,000 operating codes) than the crude criteria used by the screening tool, which is based only on 200 diagnostic and intervention groups, respectively. The divergences did not concern grouping issues but severity, investigation, or treatment strategy aspects. Finally, having the reviewers formulating an opinion in about 90% of the cases is a strong argument that even if it would be preferable to have full access to the medical records in the hospitals, partial access should not invalidate the results.

Overall, both reviewers estimated that the majority of screened stays were unnecessary: at least 86% for “unjustified stays” and at least 71% for “sometimes justified” ones (Table 1). The false positive rate for “unjustified stays” (14%) is relatively low, allowing to use this indicator to push hospitals to be rigorous in their admission criteria. The false positive rate for “sometimes justified” remain acceptable, although too high to judge the performance of hospitals. It should thus be used more parsimoniously, for instance as a basis to the reflection on how to improve the health system. As mentioned in the introduction, we did not estimate the proportion of false negative, considering that this issue could be analyzed in further research.

Our results were solely adjusted for patients’ age and types of admissions (programmed or not), adjusting for this possible source of bias. Unmeasured factors like education or social characteristics might perhaps influence the rate of unjustified stays by hospitals but whether they should be introduced into the analysis is not so clear, since improvements (e.g. specific education services or social support to outpatients) might target such determinants.

Both indicators provided precise estimations, with many hospitals having observed rates significantly above upper statistical control limits (Figs. 1 and 2).

To answer the question of the usefulness of our indicators, we first analyzed the reasons of admitting patients, even if theirs stays were a posteriori unjustified for medical reasons. A quarter of unjustified hospitalizations was due to a conservative approach adopted by clinicians to rule out a high-risk diagnosis (“suspicion of serious illness”, Table 2). A lot of these patients had diagnoses of non-specific disorders, pain, or psychiatric troubles (Additional file 2). Such stays might be avoided if accelerated diagnostic pathways were applied, as recommended for instance for chest pain [35], knowing that less than 10% of emergency department patients with chest pain are ultimately diagnosed with an acute coronary syndrome [36]. Such protocols were applied with evidence of being efficient for low-risk patients (early discharge) and high risk ones (early intervention or treatment) [37, 38]. Another frequent reason for this kind of unjustified hospitalizations is related to the lack of outpatients’ facilities to monitor serious illnesses. For instance, seizures or suspected seizures, which account for a large number of emergency admissions, might be prevented as suggested by geographical variability of admission rates [39]. Emergency care pathways might be applied to focus on rapid appointments in specialized services [40, 41]. Other non-specific complaints that could yield an unjustified stay for monitoring suspicious or serious illness include giddiness, cerebral disorders, and hypotension. Such conditions could benefit from clinical pathways’ approaches, fast access to a specialist or brain imaging to rule out a brainstem lesion.

Acute respiratory infections accounted for almost 6% of unjustified stays. We observed large variations among hospitals of admission rates for this condition, especially among young children, suggesting varying admission criteria. There is a substantial variation in the management of bronchiolitis and criteria of hospitalization or discharge to home are often subjective. Moreover, many admitted infants had no distress [42, 43], raising the question of the continuity of care between ambulatory and hospital pediatricians for instance.

The remaining causes of unnecessary stays are scarce, mostly due to contextual variables. For instance, isolation of immune-suppressed patient does not necessarily require hospital beds but might be difficult to obtain at home. Patients requiring investigation not available in ambulatory setting or living far from such infrastructure might prefer to stay in a hospital, though a hotel stay would perhaps provide the same comfort. Lack of compliance makes it difficult to find an alternative to hospitalization for instance for alcoholic, addicted people, or persons with intellectual disabilities. Care facilities at a lower level than general hospital beds, such as in nursing home, might also be offered in the proximity of patients’ home, with a supervision by their primary care physicians.

The analysis of the 820′000 hospitalizations in Swiss hospital of the period 2014-2016 showed variations of rates among hospitals. In the short term, public health services might ask hospitals not to exceed expected rates. In Switzerland, for the period 2014-2016, this would have yielded a reduction of about 6% of the hospitalizations (proportion of surplus of Table 3). To obtain a subsequent reduction of unjustified stays, several measures could be implemented, including:

- encouraging hospitals to work more closely with outpatient facilities to identify faster patients with at-risk diagnoses and provide a secured monitoring (e.g. acute coronary syndrome, epilepsy);
- providing community reinforcement of monitoring at home, implying home physician’s and nurse’s visits, education of patients and relatives;
- supporting gradual and effective treatments for pain at home;
- providing immediate home safety assessment and intervention rehabilitation to prevent dangerous situations (risk of falling, frail old patients);
- pursuing the efforts to maintain patients at home, with more specialized home nurses’ skills (IV antibiotherapy, parenteral nutrition, wound dressing or care; such services need a multidisciplinary approach to be successful [44]).

The potential of reduction of the number of hospitalizations is substantial. Considering that about 18% of them were screened “unjustified”, from which 86% were considered as unnecessary, and about 24% were screened “sometimes justified”, from which 70% were deemed unnecessary, this yields a theoretical reduction target of approximately one third. Therefore, the short-term reduction (i.e. 6%) represents only 20% of the total potential reduction.

In practice, this proportion should be considered as overuse only if less intensive care can provide similar outcomes. The question therefore arises whether it would be possible to reduce the number of hospitalizations without endangering patient safety and to what extent alternative inpatient care strategies should be tailored.

Summarizing these results, we can conclude that hospitals might be able to influence the outcome and achieve a 6% hospitalizations reduction by themselves. The analysis however also provided some evidence that involving ambulatory care facilities would be necessary to achieve a more substantial ambulatory shift (up to 24% additional reduction of the number of hospitalizations).

When using those indicators, one should be aware of the possible unintended adverse consequences. Justifying hospital stays based only on medical criteria can indeed lead to a possible harmful effect. Social or compassion care might be indicated if the hospital is the only place to shelter or surround a patient. Then, the medical justification of a stay is made a posteriori, without information about possible diagnoses considered at admission that might have justified a hospitalization. In addition, the accuracy of the indicator depends on the coding quality. For instance, if a severe acute respiratory insufficiency occurring during an influenza episode was not coded, the corresponding stay would wrongly be considered unnecessary. We therefore recommend analyzing the results carefully to see if a suboptimal coding quality might explain high rates. Finally, we discourage using these indicators to refuse funding of unjustified stays, since this could affect the security of care. Financial penalties might perhaps be used to encourage hospitals reaching the expected rates, but only globally (not for specific stays). Nevertheless, it must be kept in mind that ambulatory care also generates costs and that difficulties to improve the appropriateness of hospitalizations might also be related to regional aspects, such as insufficient ambulatory coverage.

Although the frontier between ambulatory and hospital care is not universal, we believe that this study might be replicated and applied in other countries.

Other classifications’ tools could be used, given that co-morbidities are explicitly reflected and that diagnoses and intervention categories are sufficiently homogeneous to determine whether they justify hospitalizations. Some authors will probably propose refinements or adaptations (for instance, we had some difficulties to decide whether elective alcoholic withdrawals or non-traumatic painful back might justify hospitalizations).

Further research should focus on the pediatric context to better understand interregional practice differences. Analyzing unjustified stays from detailed medical records is necessary to understand what kind of ambulatory care is missing to ensure secure alternatives to hospitalization. However, we believe that our results are interesting since they show that there is a substantial potential to shift toward ambulatory care in Medicine and Pediatrics and enable to delineate the most promising domains. In this respect, both indicators might be used for planning purpose.

## Conclusion

We recommend using the “unjustified stays” and “sometimes justified stays” indicators to monitor inappropriate hospitalizations. Based on these two indicators, we found that one third of the medicine and pediatric hospitalizations made in Switzerland between 2014 and 2016 did not have a clear a-posteriori medical justification. Nevertheless, our results suggest that only a part of these stays (6% of eligible stays) could be avoided without changes in the health care system. To obtain a more substantial reduction whilst ensuring patients’ safety, measures to reinforce ambulatory care are required.

## Supplementary Information

**Additional file 1.** List of diagnostic categories (SQLape®).

**Additional file 2.** Most frequent diagnoses categories (all Swiss hospitals, 2016).

## Data Availability

The data that support the findings of this study are available from the Swiss *Federal Statistical Office* but restrictions apply to the availability and use of these data. Data are however available from the first author upon reasonable request and with permission of the Federal Statistical Office.
