# Supplementary material for: Measuring medically unjustified hospitalizations in Switzerland
Source: BMC Health Serv Res. 2022 Feb 7;22:158. doi: 10.1186/s12913-022-07569-3 (PMC8822832; doi:10.1186/s12913-022-07569-3)
Supplement: Supplementary file 1 — Additional file 1. List of diagnostic categories (SQLape®). [file 12913_2022_7569_MOESM1_ESM.docx]

Additional file 1. List of diagnostic categories (SQLape®)

| System | SQLape® category | | Hospitalization |
| --- | --- | --- | --- |
| Newborn | F-pA | Delivery | almost always |
| Newborn | F-pP | Pre- or post-partum problem | almost always |
| Newborn | F-pV | Abortion | Sometimes |
| Newborn | F-pG | Pregnancy | Sometimes |
| Newborn | B-nE | Newborn, weight 600-999 g | almost always |
| newborn | B-nD | Newborn, weight 1000-1499 g | almost always |
| newborn | B-nC | Newborn, weight 1500-1999 g | almost always |
| newborn | B-nB | Newborn, weight 2000-2199 g | almost always |
| newborn | B+nZ | Newborn with major disorder | almost always |
| newborn | B-nZ | Newborn with minor disorder | almost always |
| newborn | B-zZ | New born dead on first day | almost always |
| newborn | B-aC | Cardiac congenital malformation, newborn | almost always |
| newborn | B-nA | Healthy newborn | almost always |
| circulatory | C+sC | Acute myocardial infarction | almost always |
| circulatory | C-sC | Other acute ischemic heart disease | almost always |
| circulatory | C-sV | Gangrene | almost always |
| circulatory | C-oC | Occlusion of cerebral or precerebral artery | almost always |
| circulatory | C-oV | Thrombosis or embolism of limb | Sometimes |
| circulatory | C-oG | Other thrombosis or embolism | almost always |
| circulatory | C-tC | Heart or large vessel injury | almost always |
| circulatory | C-iC | Heart inflammation | almost always |
| circulatory | C-aC | Cardiac congenital malformation, except newborn | Sometimes |
| circulatory | C-fC | Collapse | almost always |
| circulatory | C+fV | Severe hypertension | almost always |
| circulatory | C-dC | Heart failure | sometimes |
| circulatory | C-dH | Ruptured aneurism of large vessel | almost always |
| circulatory | C-dG | Other aneurism | sometimes |
| circulatory | C-zA | Cardiac dysrhythmia | sometimes |
| circulatory | C-zG | Other disease of large vessels | sometimes |
| circulatory | C-zV | Other disease of vessels | sometimes |
| circulatory | C-sA | Coronary arteries disorder | almost never |
| circulatory | C-zS | Other circulatory disorder | almost never |
| circulatory | C-fV | Non-severe hypertension | almost never |
| respiratory | R-sP | Pulmonary embolism | almost always |
| respiratory | R-tT | Chest injury | almost always |
| respiratory | R-iZ | Influenza or acute bronchitis | almost never |
| respiratory | R-iB | Chronic bronchitis and asthma | almost never |
| respiratory | R-iP | Other pulmonary infection | sometimes |
| respiratory | R-mP | Malignant neoplasm, lung | almost never |
| respiratory | R-fR | Acute respiratory failure | almost always |
| respiratory | R-dP | Interstitial pulmonary disease | sometimes |
| respiratory | R-dR | Chronic respiratory failure | sometimes |
| respiratory | R-zZ | Other pulmonary disease | sometimes |
| respiratory | R-zR | Other respiratory disorder | almost never |
| nervous | N-sC | Cerebrovascular accident | almost always |
| nervous | N-sT | Transient cerebral ischemia | almost always |
| nervous | N-oC | Hydrocephalus | sometimes |
| nervous | N-oR | Compression of spinal nerve root | almost never |
| nervous | N+tC | Major intracranial injury | almost always |
| nervous | N-tC | Minor cerebral injury | almost always |
| nervous | N-iC | Meningitis or encephalitis | almost always |
| nervous | N-iM | Guillain-Barre syndrome | almost always |
| nervous | N-iS | Multiple sclerosis | almost never |
| nervous | N-hC | Non-traumatic cerebral hemorrhage | almost always |
| nervous | N-mC | Malignant neoplasm, brain | almost never |
| nervous | N-mN | Neoplasm of nerve | almost never |
| nervous | N-bC | Benign neoplasm of brain | almost never |
| nervous | N-dP | Parkinson's disease | almost never |
| nervous | N-dM | Extended paralysis | sometimes |
| nervous | N-dC | Degenerative disease of brain | almost never |
| nervous | N-fE | Epilepsy | almost never |
| nervous | N-fM | Migraine | almost never |
| nervous | N-fN | Neuralgia | almost never |
| nervous | N-zC | Other disease of the brain | almost always |
| nervous | N-zS | Cerebral disorder | almost never |
| nervous | N-zM | Disease of the spinal cord | sometimes |
| urinary | U-oU | Urinary tract obstruction | sometimes |
| urinary | U+iU | Major urinary infection | sometimes |
| urinary | U-iU | Minor urinary infection | almost never |
| urinary | U-mU | Malignant neoplasm, urinary organ | almost never |
| urinary | U-bP | Prostatic hyperplasia | almost never |
| urinary | U-fR | Acute nephropathy | almost always |
| urinary | U-dR | Chronic nephropathy | sometimes |
| urinary | U-dT | End stage renal disease | sometimes |
| urinary | U-zV | Bladder disease | sometimes |
| urinary | U-zU | Other urinary disease | almost never |
| digestive | D-oI | Intestinal obstruction | almost always |
| digestive | D-iP | Peritonitis | almost always |
| digestive | D+iI | Complicated gastrointestinal ulcer | almost always |
| digestive | D-iI | Gastroenteritis or gastrointestinal ulcer | almost never |
| digestive | D-iC | Inflammatory entero-colitis | sometimes |
| digestive | D-iD | Gastrointestinal specified infection | sometimes |
| digestive | D-mS | Malignant neoplasm of stomach | almost never |
| digestive | D-mI | Malignant neoplasm of intestine | almost never |
| digestive | D-mR | Malignant neoplasm of anus or rectum | almost never |
| digestive | D-dI | Intestine diverticula with complication or fistula | almost always |
| digestive | D-dD | Gastrointestinal haemorrhage | almost always |
| digestive | D-fZ | Functional digestive disorder | almost never |
| digestive | D-zH | Uncomplicated hernia | sometimes |
| digestive | D-zO | Other disease of esophagus | almost never |
| digestive | D-zT | Other disease of small intestine | sometimes |
| digestive | D-zZ | Other digestive disease | sometimes |
| hepatic | H-oB | Biliary obtruction without complication | almost never |
| hepatic | H-iH | Hepatitis | almost never |
| hepatic | H-iP | Acute pancreatitis | almost always |
| hepatic | H-iB | Cholecystitis or angiocholitis | almost always |
| hepatic | H-mH | Malignant neoplasm of the liver | almost never |
| hepatic | H-mP | Malignant neoplasm of the pancreas | almost never |
| hepatic | H-dH | Liver cirrhosis | almost never |
| hepatic | H-dP | Chronic pancreatitis | sometimes |
| hepatic | H-fB | Other biliary disorder | almost never |
| hepatic | H-zH | Other liver disease | almost always |
| endocrine | E+dD | Diabetes with coma or ketoacidosis | almost always |
| endocrine | E-dD | Diabetes without complication | almost never |
| endocrine | E-fT | Thyroid disorders | almost never |
| endocrine | E-zS | Other endocrine disease | almost never |
| blood | S-iS | Sepsis | almost always |
| blood | S+iZ | Severe infection | almost always |
| blood | S-iZ | Bacterial infection | sometimes |
| blood | S-mM | Acute myeloid leukemia | almost never |
| blood | S-mL | Acute lymphoid leukemia | almost never |
| blood | S-mS | Lymphoma, other hematopoietic neoplasm | almost never |
| blood | S-mO | Secondary malignant neoplasm, bone | almost never |
| blood | S-dL | Agranulocytosis | almost always |
| blood | S-dS | Cachexia | sometimes |
| blood | S+dI | Acquired immunodeficency syndrome | almost never |
| blood | S-dI | Other immune disorder | almost never |
| blood | S-fT | Disseminated intravascular coagulopathy | almost always |
| blood | S-fA | Deficiency anemia | almost never |
| blood | S-zA | Other anemia | almost never |
| blood | S-zT | Other coagulation disorder | sometimes |
| blood | S-fZ | Metabolic or blood disorder | almost never |
| blood | S-fM | Metabolic disease or general symptoms | sometimes |
| blood | S-fL | Hyperlipidemia | almost never |
| locomotion | L-tT | Skull injury | almost always |
| locomotion | L-tV | Vertebral column injury | almost always |
| locomotion | L-tC | Fracture of pelvis | sometimes |
| locomotion | L-tB | Arm and forearm injury | almost never |
| locomotion | L-tJ | Leg injury | almost never |
| locomotion | L-tL | Other muskuloskeletal injury | almost never |
| locomotion | L-tZ | Other severe injury | almost always |
| locomotion | L-iO | Musculoskeletal infection | almost always |
| locomotion | L+iL | Severe musculoskeletal inflammation | sometimes |
| locomotion | L-iL | Polyarthritis and other inflammation | almost never |
| locomotion | L-iG | Gout and secondary arthropathies | almost never |
| locomotion | L+dO | Severe degenerative disease of bone | sometimes |
| locomotion | L-dV | Degenerative disease of vertebra | sometimes |
| locomotion | L-dG | Degenerative disease of knee | almost never |
| locomotion | L-dO | Degenerative disease of other bones | almost never |
| locomotion | L-zC | Other spinal disease | sometimes |
| locomotion | L-fV | Back pain | almost never |
| locomotion | L-zM | Muscular disease | almost never |
| female | F-iO | Infection of the ovary | sometimes |
| female | F-iZ | Other infection of female organ | almost never |
| female | F-mG | Malignant neoplasm of genital organs | almost never |
| female | F-mM | Malignant neoplasm of the breast | almost never |
| female | F-bZ | Benign neoplasm of female organ | almost never |
| female | F-zZ | Other female disease | almost never |
| cutaneous | T+tT | Third degree or extended burns | almost always |
| cutaneous | T-tT | Open wound and non-superficial burns | sometimes |
| cutaneous | T-tS | Superficial injury | almost never |
| cutaneous | T-iB | Extended bullous dermatose | almost always |
| cutaneous | T+iS | Skin abscess | sometimes |
| cutaneous | T-iS | Localised skin infection | almost never |
| cutaneous | T-iT | Inflammatory dermatitis | almost never |
| cutaneous | T-mS | Malignant neoplasm, skin | almost never |
| cutaneous | T-dS | Skin ulcer | sometimes |
| cutaneous | T-dD | Decubitus ulcer | sometimes |
| cutaneous | T-zZ | Other skin disease | almost never |
| cutaneous | T-zT | Obesity | almost never |
| ocular | Y-iO | Eye infection | sometimes |
| ocular | Y-tO | Eye injury | sometimes |
| ocular | Y-dO | Glaucoma | almost never |
| ocular | Y-zZ | Disease of anterior chamber | almost never |
| ocular | Y-zP | Disease of posterior chamber | sometimes |
| ORL | O+iO | Severe upper respiratory infection | almost always |
| ORL | O-iO | Other upper respiratory infection | almost never |
| ORL | O-mO | Malignant neoplasm, upper respiratory | almost never |
| ORL | O-zV | Giddiness and deafness | almost never |
| psychic | P-tD | Depression | almost never |
| psychic | P-fH | Psychosis, hallucination and delirium | sometimes |
| psychic | P-dZ | Dementia | almost never |
| psychic | P-zA | Anorexia nervosa | sometimes |
| psychic | P-xA | Alcohol abuse, uncomplicated | almost never |
| psychic | P-xS | Other substance abuse, uncomplicated | almost never |
| psychic | P+xS | Substance abuse, complicated | almost always |
| psychic | P-zZ | Other psychiatric disorder | almost never |
| other | Z-zG | Transplant rejection | almost always |
| other | Z+zX | Poisoning | sometimes |
| other | Z-tZ | Injury of other internal organ | almost always |
| other | Z-mC | Chemotherapy | almost never |
| other | Z-mM | Secondary malignant neoplasm, except bone | almost never |
| other | Z-mR | Radiotherapy | almost never |
| other | Z-mZ | Other malignant neoplasm | almost never |
| other | Z-zR | Rehabilitation or palliative care | sometimes |
| other | Z-zD | Pain | almost never |
| other | Z-iZ | Infection, not classified elsewhere | almost never |
| other | Z-zX | Accident | almost never |
| other | Z-zP | Transplant recipient | almost never |
| other | Z-zZ | Other disorders | almost never |
| other | Z-fD | Organ donation | almost always |
| other | Z-zM | Without valid information | almost never |
| other | Z-cT | Decubitus and thrombo-embolic complications | almost never |
