# Supplementary material for: Measuring medically unjustified hospitalizations in Switzerland
Source: BMC Health Serv Res. 2022 Feb 7;22:158. doi: 10.1186/s12913-022-07569-3 (PMC8822832; doi:10.1186/s12913-022-07569-3)
Supplement: Supplementary file 2 — Additional file 2. Most frequent diagnoses categories (all Swiss hospitals, 2016). [file 12913_2022_7569_MOESM2_ESM.docx]

Additional file 2. Most frequent diagnoses categories (all Swiss hospitals, 2016)

| Unjustified stays | Frequencies |  | Sometimes justified stays | Frequencies |
| --- | --- | --- | --- | --- |
| Other psychiatric disorder | 12.9% |  | Cardiac dysrhythmia | 12.6% |
| Non-severe hyper-/hypotension | 6.9% |  | Metabolic disease, general sympt. | 12.3% |
| Not specific disorders | 5.2% |  | Other pulmonary infection | 10.0% |
| Pain | 4.5% |  | Gastrointestinal specified infection | 8.5% |
| Cerebral disorder | 3.8% |  | Urinary tract obstruction | 5.5% |
| Influenza or acute bronchitis | 3.5% |  | Chronic nephropathy | 5.3% |
| Depression | 3.4% |  | Bacterial infection | 5.2% |
| Epilepsy | 3.3% |  | Pregnancy | 4.6% |
| Diabetes without complication | 3.2% |  | Skin abscess | 4.3% |
| Back pain | 2.7% |  | Degenerative disease of vertebra | 3.6% |
| Other muskuloskeletal injury | 2.5% |  | Heart failure | 3.1% |
| Superficial injury | 2.3% |  | Open wound, not superficial burns | 2.3% |
| Other upper respiratory infection | 2.2% |  | Other spinal disease | 2.0% |
| Other circulatory disorder | 2.2% |  | Fracture of pelvis | 1.7% |
| Other female disease | 1.8% |  | Chronic respiratory failure | 1.4% |
| Infection, other | 1.8% |  | Inflammatory entero-colitis | 1.3% |
| Coronary arteries disorder | 1.6% |  | Disease of the spinal cord | 1.3% |
| Endoscopy | 1.6% |  | Bones severe degenerative disease | 1.2% |
| Metabolic or blood disorder | 1.5% |  | Skin ulcer | 1.2% |
| Thyroid disorders | 1.4% |  | Poisoning | 1.2% |
| Other respiratory disorder | 1.4% |  | Other coagulation disorder | 1.1% |
| Chronic bronchitis and asthma | 1.4% |  | Cachexia | 1.0% |
| Functional digestive disorder | 1.3% |  | Extended paralysis | 0.8% |
| Compression of spinal nerve root | 1.2% |  | Severe musculoskeletal inflam. | 0.7% |
| Giddiness and deafness | 1.1% |  | Thrombosis or embolism of limb | 0.7% |
| Degenerative disease of brain | 1.1% |  | Decubitus, thromboemb. compl. | 0.7% |
| Gastroenteritis/intestinal ulcer | 1.1% |  | Interstitial pulmonary disease | 0.7% |
| Other disease of esophagus | 1.0% |  | Eye injury | 0.5% |
| Hepatitis | 1.0% |  | Other digestive disease | 0.5% |
| Inflammatory dermatitis | 1.0% |  | Other aneurism | 0.5% |
| Liver cirrhosis | 1.0% |  | Bladder disease | 0.5% |
| Biliary obtruction without complic. | 0.9% |  | Other disease of vessels | 0.4% |
| Other skin disease | 0.9% |  | Psychosis, delirium | 0.4% |
| Localised skin infection | 0.9% |  | Other pulmonary disease | 0.3% |
| Polyarthritis | 0.8% |  | Anorexia nervosa | 0.3% |
| Alcohol abuse, uncomplicated | 0.8% |  | Cardiac congen. malformation | 0.3% |
| Leg injury | 0.8% |  | Eye infection | 0.3% |
| Arthroscopy or arthrocentesis | 0.7% |  | Uncomplicated hernia | 0.2% |
| Secondary malignant neoplasm | 0.7% |  | Hydrocephalus | 0.2% |
| Migraine | 0.7% |  | Rehabilitation or palliative care | 0.2% |
| Lymphoma, other neoplasm | 0.6% |  | Chronic pancreatitis | 0.2% |
| Malignant neoplasm, urinary | 0.6% |  | Disease of posterior chamber | 0.2% |
| Arm and forearm injury | 0.6% |  | End stage renal disease | 0.2% |
| Other diseases (35 categories) | 10.3% |  | Other diseases (4 categories) | 0.6% |
| Total | 100.0% |  | Total | 100.0% |
